# Conjoined twins and conjoined triplets: At the heart of the matter

**DOI:** 10.1002/bdr2.2066

**Published:** 2022-06-29

**Authors:** Roelof‐Jan Oostra, Annelieke N. Schepens‐Franke, Giovanni Magno, Alberto Zanatta, Lucas L. Boer

**Affiliations:** ^1^ Department of Medical Biology, Section Clinical Anatomy & Embryology, Amsterdam University Medical Centers, location Academic Medical Center University of Amsterdam The Netherlands; ^2^ Department of Imaging, Section Anatomy and Museum for Anatomy and Pathology Radboud University Medical Center Nijmegen The Netherlands; ^3^ Department of Cardiac, Thoracic and Vascular Sciences, Section of Medical Humanities, Padua University Medical School and Museum of Pathological Anatomy University Museums Centre, University of Padua Padua Italy

**Keywords:** congenital anomaly, conjoined triplets, conjoined twins, embryonic disk model, embryology, heart, teratology

## Abstract

Conjoined triplets are among the rarest of human malformations, as are asymmetric or parasitic conjoined twins. Based on a very modest corpus of recent literature, we applied the embryonic disk model of conjoined twinning to 10 previously reported cases involving asymmetric anatomical multiplications to determine whether they concerned conjoined twins or conjoined triplets. In spite of their phenotypic similarities, we diagnosed four of these cases as conjoined twins and three of them as conjoined triplets. In the remaining three cases, no definite diagnosis could be made, as essential information was lacking from the reports. We conclude that it is not necessarily the expected duplication or triplication of structures that points to the correct diagnosis in these cases, but the number and mutual position of the hearts they presented with. Considering their rarity we stress to thoroughly investigate and describe internal (dys)morphology in novel cases of (asymmetric) conjoined twins and triplets to further unravel their pathogenicity and come to the correct diagnoses.

## 1 CONJOINED TWINS, TRIPLETS, AND (A)SYMMETRY

**FIGURE 1:**
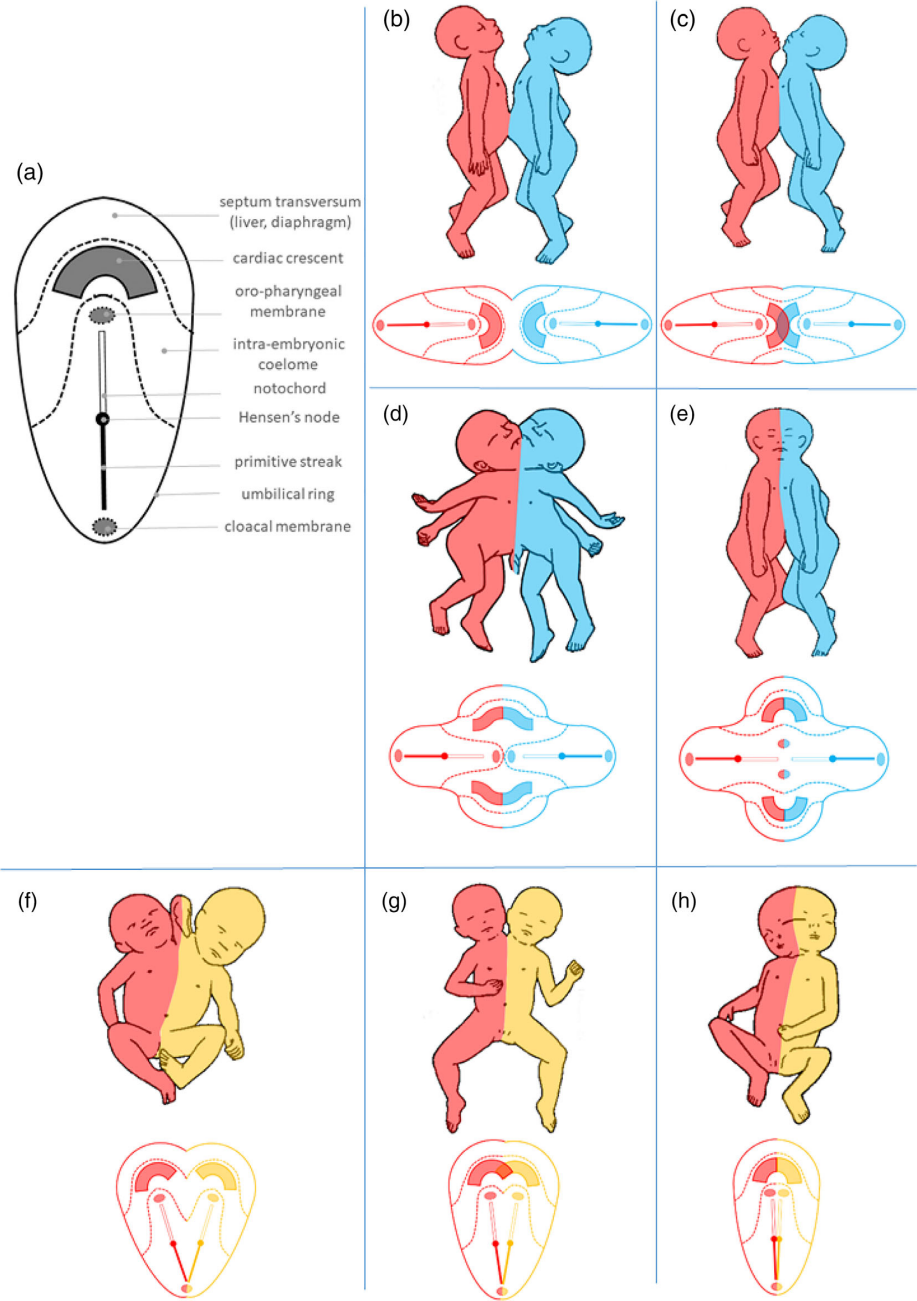
The embryonic disk model applied to ventral and lateral conjoined twinning. (a) Schematic representation of the embryonic disks as it develops during gastrulation, prior to the folding process that gives rise to the primordial embryonic body. (b–e) In ventral conjunction, the initial distance between the antero‐posteriorly opposing disk primordia determines to what extend the bodies of the twins shared and subsequently become involved in the process of neo‐axial orientation, which leads to compound structures and organs. (f–h) In lateral conjunction, the disk primordia are positioned next to each other and their initial distance and the angle between their longitudinal axes determines the extend of interaction aplasia, which causes adjacent parts of both bodies not to be formed. See also Table 1 for details and terminology concerning the various conjunction types. Please note that the conditions mentioned here concern arbitrarily defined entities that represent recognizable patterns within a spectral continuum of conjunction types.

Conjoined twins are a rare phenomenon of unknown etiology, with a prevalence of approximately 1.5 per 100,000 births (Mutchinick et al., 2011). These twins are physically connected with each other and may share homologous tissues, organs, and even larger parts of their body. In accordance with the site of conjunction they can be grouped in ventrally, laterally, caudally, and dorsally conjoined twins, although overlap between these groups has been reported frequently (Boer, Schepens‐Franke, & Oostra, 2019; Boer, Schepens‐Franke, Winter, & Oostra, 2021; Spencer, 2003). Their pathogenesis is still disputed but can be interpreted as having resulted from two embryonic primordia―instead of one―in a single embryoblast, forming two embryonic disks in close proximity of each other (Figure 1a) (Spencer, 2003). Subsequently, the growing disks will interfere with each other's development, the type, and severity of which are determined by their mutual position and distance (Boer et al., 2019; Boer et al., 2021). Below, a brief outline is given on the mechanisms involved in conjoined twinning, to become acquainted with the theories and descriptions of duplicated and triplicated embryonic disk models as postulated in this article.

**FIGURE 2:**
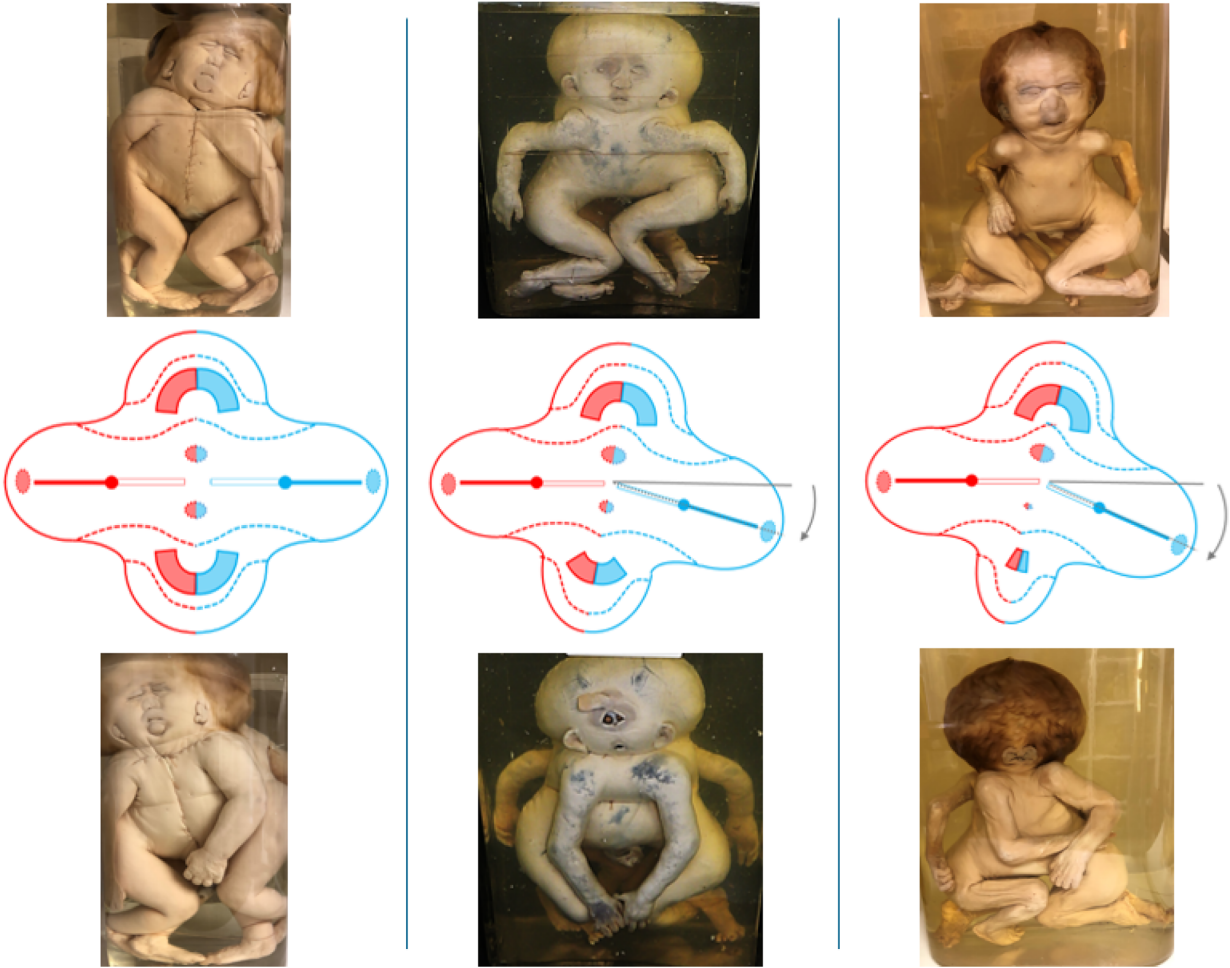
The effects of angulation between the longitudinal axes of the embryonic disks in ventral conjunction. When the longitudinal axes of the embryonic disks―involved in ventral conjunction―are not entirely parallel but more or less angulated, the opposing planes of the conjunction area attain a convex and a concave aspect, respectively. The compound structures and organs that develop in the convex plane usually have a more or less normal appearance, for example, the face in cephalothoracoileopagus (top row), whereas those developing in the concave plane are subjected to interaction aplasia, the severity of which correlates with the degree of initial angulation. In cephalothoracoileopagus, this causes the compound face in this plane to present with a phenotypic spectrum that resembles that of holoprosencephaly (bottom row). *Source*: Specimens from the *Narrenturm* collection in Vienna (Austria)

In ventrally conjoined twins, the embryonic disks are connected in their anterior aspects. With increasing intimacy, this leads to shared umbilical, abdominal, thoracic, and even craniofacial structures. A typical phenomenon in ventral conjunction―known as neo‐axial orientation―is the formation of compound structures and organs, built up of divided and diverted organ primordia of both twins, in a plane perpendicular to their original orientation (Figure 1b–e and Table 1). In laterally conjoined twins, the two disks are positioned side by side. Depending on their mutual distance, this causes interaction aplasia, in which parts of both bodies that present in the plane of conjunction will not be formed (Figure 1f–h and Table 1). Interaction aplasia also occurs in ventrally conjoined twins when the longitudinal axes of the embryonic disks are angulated instead of parallel. This concerns the neo‐axially oriented compound structures at the resulting concave side of the conjunction area (Figure 2).

**TABLE 1:** Types of conjoined twinning presented in this article and their characteristics

Ventral conjunction	Omphalopagus	Joined at the sternal ends, diaphragms, livers and umbilicus; two separate hearts (Figure 1b)
	Thoracoileopagus	Joined at the thoraxes and umbilicus; two compound sternums, diaphragms and livers; one shared multichambered heart (Figure 1c)
	Prosopothoracoileopagus	Joined at the lower faces, necks, thoraxes and umbilicus; usually two compound hearts, sternums, diaphragms, and livers (Figure 1d)
	Cephalothoracoileopagus	Joined at the heads, necks, thoraxes and umbilicus; two compound hearts, faces, sternums, diaphragms, and livers (Figure 1e)
Lateral conjunction	Parapagus dicephalus tribrachius	Shared lower body and abdomen; partially duplicated thorax; two heads and a shared third arm; usually one shared multichambered heart or two separate hearts (Figure 1f)
	Parapagus dicephalus dibrachius	Shared lower and upper body; two heads; usually one shared multichambered or four‐chambered heart (Figure 1g)
	Parapagus diprosopus	Shared body and head; (partially) duplicated face; usually one shared four‐chambered heart (Figure 1h)

Asymmetric conjoined twinning occurs approximately 1 in 1,000,000 live births and refers to a condition in which one of the twins is markedly less well developed compared to the other. It is generally assumed to result from the same developmental process that gives rise to symmetric conjoined twins, but it is complicated by either an imbalance in embryonic disk size or a disruptive incident that causes a delay or an arrest of development in one of the twins (Spencer, 2001). The affected twin may present with a wide variety of more or less recognizable structures, organs, and limbs but nearly always lacks a functioning heart, which is why it is referred to as “parasite,” since its life seems to be supported by the host twin or “autosite.” Asymmetric twinning has been reported in most conjunction types, albeit with markedly different frequencies (Spencer, 2001). It should be stressed here that the term “asymmetry” has also been in use to refer to the interaction aplasia in ventrally conjoined twins with angulated longitudinal axes, especially where it concerns the developmental discrepancies in the compound faces of cephalothoracoileopagus (see Figure 2).

Among the rarest expressions of any twin‐related anomalies are conjoined triplets. This term refers to a condition in which a single organic entity consists of three identifiable (but not necessarily symmetrically represented) individuals. As such, it should be differentiated from triplets that consist of conjoined twins and a singleton sib, which although rare in itself, is being reported around once a year in the scientific literature. In addition to some historical descriptions, only three attested clinical cases of symmetric conjoined triplets have been published to date (Athanasiadis, Tzannatos, Mikos, Zafrakas, & Bontis, 2005; Reina, 1841; Rode et al., 2006), which will be discussed in detail below.

In this article, we will revisit seven previously reported cases of asymmetric multiplications, to demonstrate: (a) that, although conjoined twins and conjoined triplets are distinctly discernable conditions, the phenotypic characteristics in the presented cases initially suggest both diagnoses to be applicable, and (b) that the key to the correct diagnosis is to be found in the topography of the heart(s) and not per se in the duplication or triplication of structures which stresses the importance of detailed morphological examination. Finally, this approach demonstrates that previously published cases can be (re)diagnosed when known patterns of conjoined twinning and conjoined triplets with their accompanying embryonic disk models are extrapolated.

### 1.1 Asymmetric cephalothoracoileopagus twins and their embryonic disk models

Asymmetric conjoined twinning has been extensively reviewed by Spencer (Spencer, 2001) and Sharma (Sharma, Mobin, Lypka, & Urata, 2010); together covering almost 200 cases in a 135 years period. The most common location of autosite‐parasite conjunction―accounting for almost 60% of the cases―is ventrally, between the abdomens, which is comparable to the percentage of *omphalopagus* in symmetric conjoined twins (Spencer, 2001, 2003). The degree of parasite organization and development is very variable and ranges from small ill‐defined tissue lumps to well‐recognizable body parts such as extremities, up to more or less complete but malformed bodies, which potentially can make highly developed parasites difficult to distinguish from discordancy for malformations in otherwise symmetric conjoined twins. This variety is probably caused not only by the site and intensity of the conjunction but also by the timing and effect of the incident that triggered developmental arrest in the parasite. However, the presence of cardiac tissue in the parasite has hardly ever been reported, which makes the following two case reports―that were not included in the review by Sharma et al., nor was their type of conjunction―exceptional.

**FIGURE 3:**
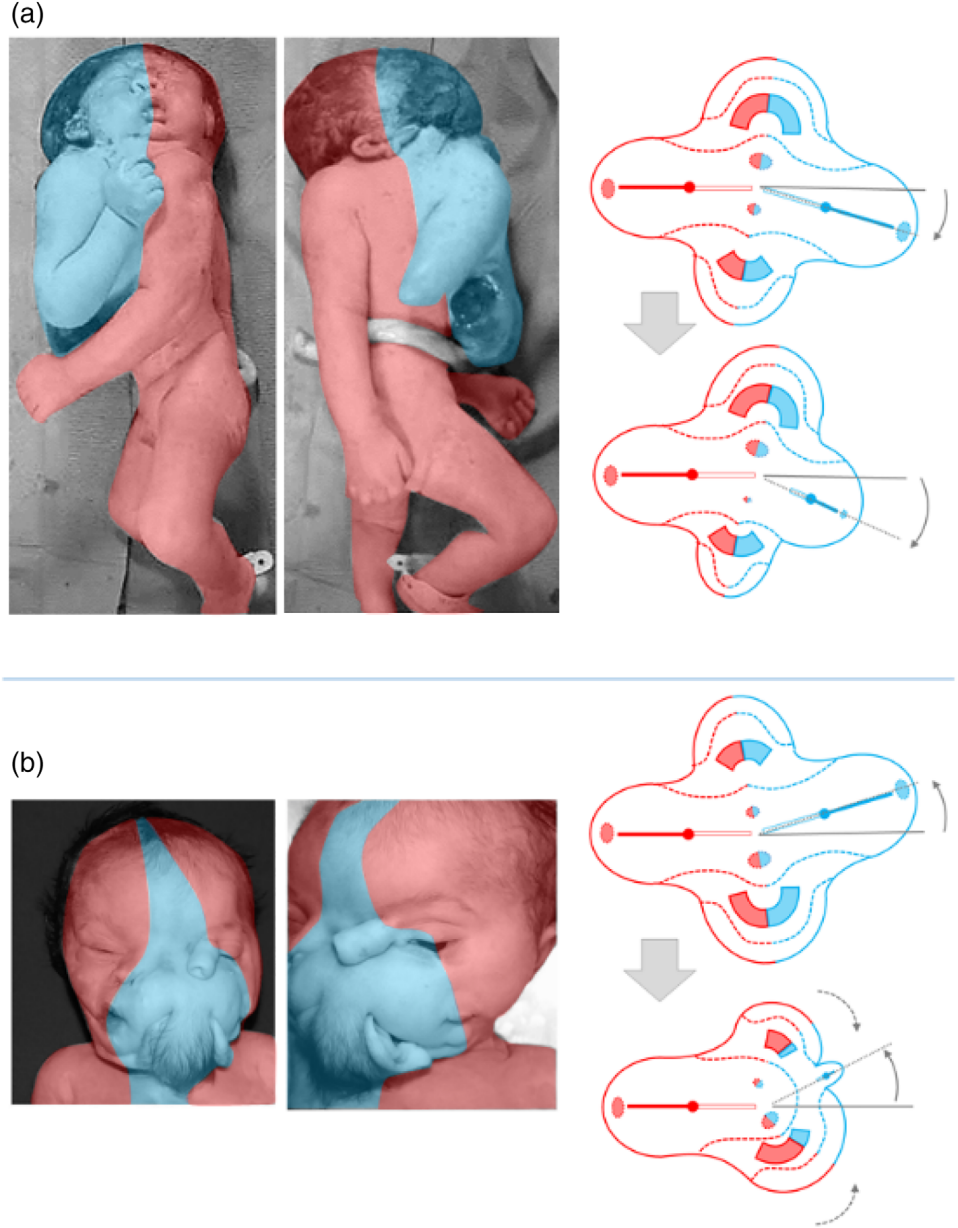
Asymmetric cephalothoracoileopagus. (a) Case described by Kaufmann (Kaufmann et al., 2007) including schematics of the embryonic disk model before (top) and after parasitic regression (bottom). *Source*: Reprinted with permission from TheFetus.net. (b) Case described by Kastenbaum (Kastenbaum, McPherson, Murdoch, & Ozolek, 2009) including schematics of the embryonic disk model before (top) and after parasitic regression (bottom). As a result of the profound regression, the neo‐axial orientation of compound structures and organs in the convex and concave conjunction planes is largely abolished (dashed arrows). *Source*: Used with permission from Sage journals

Kaufmann (Kaufmann, Comalli Dillon, Cuillier, Lafitte, & Ranjatoelina, 2007) described a case of a monochorionic diamniotic triplet, born at 34 weeks of gestation after caesarian section. Already at 17 weeks of gestation, ultrasonography (US) indicated the presence of one normal and one malformed fetus. A second US scan at 25 weeks of gestation revealed that the malformed fetus had, two hearts, two stomachs, one normal and one truncated vertebral column, profound craniofacial and cerebral abnormalities, an occipital meningocele, an omphalocele and abnormal extremities. A triplet pregnancy with one normal fetus and one set of conjoined twins was suspected and confirmed after delivery and subsequent postmortem investigation. The conjoined twins had a single head with two opposing faces, one fairly well shaped, the other one reduced to a proboscis and two ears. A normally formed body was ventrally attached at the head, thorax and abdomen, to a severely malformed fetus, that lacked a pelvis and lower extremities, and featured the prenatally assessed occipital meningocele and omphalocele, and a peromelic arm. The two hearts of these twins both showed profound septal and conotruncal malformations. This case is to be diagnosed as asymmetric *cephalothoracoileopagus* conjoined twins with axial angulation, causing profound midfacial hypoplasia in one of the compound faces (Figure 3a). The involution of the parasite mainly concerned its lower body half and bears resemblance with only two other cases (Bordenave, 1776; Klein, 1818) (see Supplementary data S1). The presence of two hearts, contributed to by both the autosite and the parasite, is in accordance with the embryonic disk model for this type of conjoined twinning.

Kastenbaum (Kastenbaum et al., 2009) reported on a male infant with prenatally assessed severe craniofacial and cardiac malformations, born spontaneously at almost 41 weeks of gestation, that lived for about a week. The head presented with two faces, separated by a hair‐covered tissue mass that was flanked by two dysplastic ears. The right sided face had a nose, a mouth, a single eye, and an ear on the right side, all normally shaped. The left sided face had a central eye, a proboscis placed above it, a hypoplastic mouth, and a normally shaped left sided ear. In between the nose and the proboscis, a dimple was noted, without recognizable ocular structures. Apart from widely spaced nipples and two unexplained depressions near the midline external examination of the trunk and extremities showed no abnormalities. At autopsy, the tissue mass in between the two faces appeared to contain rudiments of cranial bones, meninges and neural tissue. In addition to two normal lungs, the thorax of the infant was found to contain two hearts within a single pericardial cavity. The left‐sided heart had about one third of the size of the right‐sided heart. Both hearts had severe conotruncal malformations. All abdominal organs showed normal anatomy. Despite the profoundly different presentation, compared to the case described by Kaufmann (Kaufmann et al., 2007) this too concerns asymmetric *cephalothoracoileopagus* conjoined twins with axial angulation. However, in this case, the parasite is regressed to such an extent that the neo‐axial orientation of the compound faces and hearts is largely abolished. As a result, their orientation is almost side‐by‐side instead of opposing, with the hearts enclosed by a single pericardial cavity; this is confirmed by the embryonic disk model (Figure 3b). It deserves mentioning that the authors of this case report also arrived at this diagnosis (Kastenbaum et al., 2009).

### 1.2 Conjoined triplets and their embryonic disk models

**FIGURE 4:**
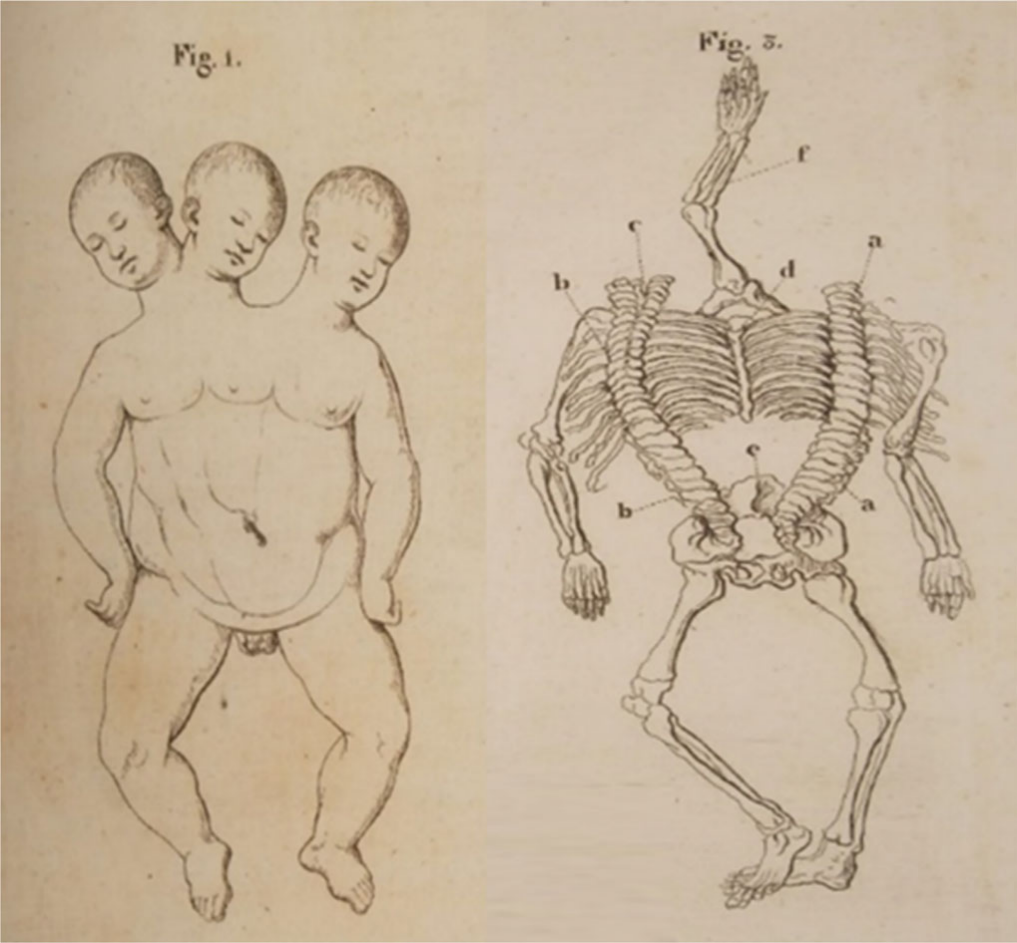
Lithograph of a tricephalic fetus (Reina, 1841)

**FIGURE 5:**
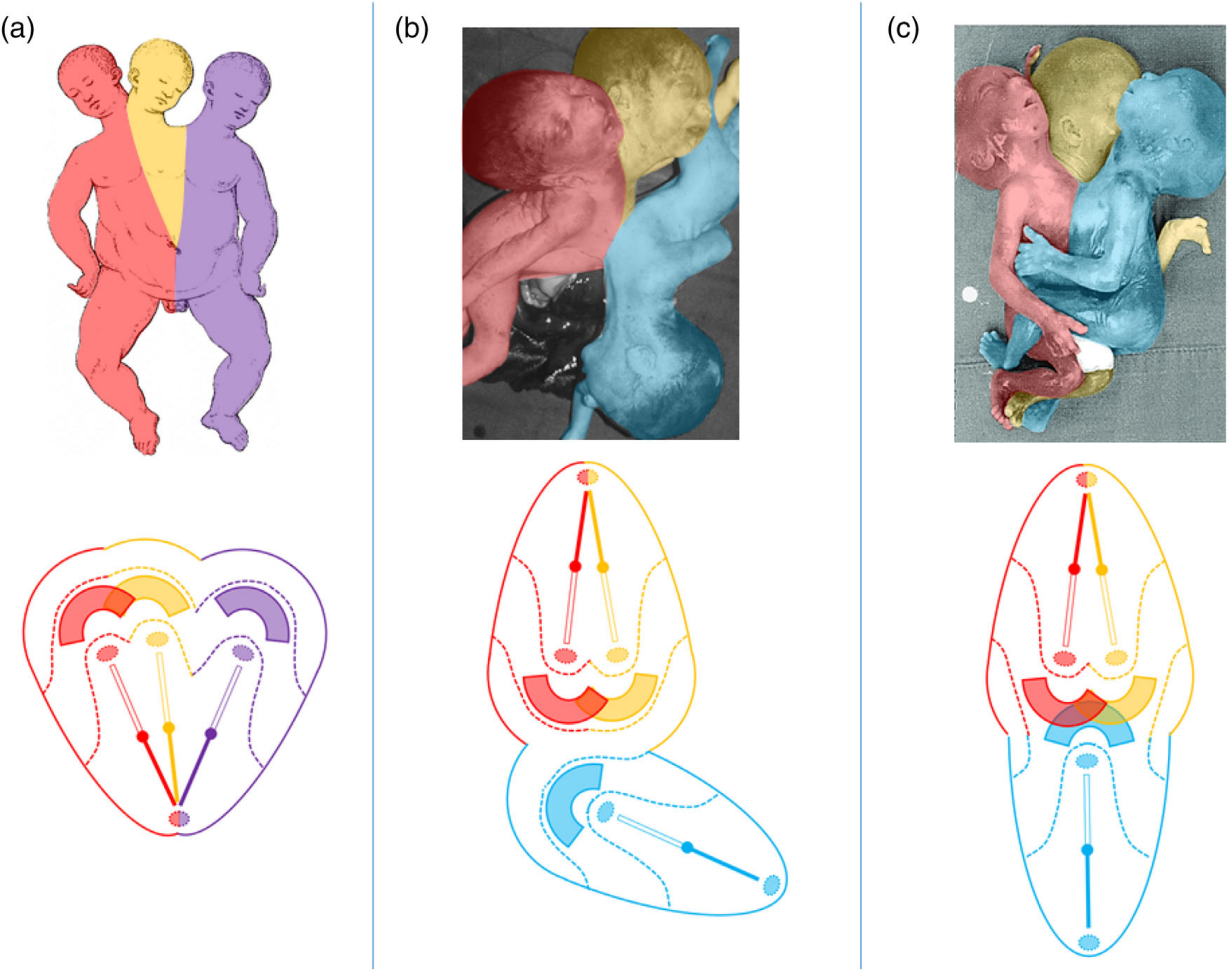
The embryonic disk model applied to symmetric conjoined triplets. (a) Case described by Reina of a parapagus dicephalus dibrachius‐parapagus dicephalus tribrachius conjoined triplet (Reina, 1841). (b) Case described by Athanasiadis et al. (2005)) of a parapagus dicephalus dibrachius‐omphalopagus conjoined triplet. *Source*: Photograph used with permission from the *American Journal of Obstetrics and Gynecology* (Athanasiadis et al., 2005). (c) Case described by Rode et al. (2006) of a parapagus dicephalus tribrachius‐thoracoileopagus conjoined triplet. *Source*: Photograph used with permission from Rode et al. (Rode et al., 2006)

Symmetric conjoined triplets are likely to be among the rarest of human malformations. One of the most convincing cases of symmetric conjoined triplets is that of Euplio Reina presented in his inaugural lecture in 1832 at the University of Catania, Sicily, and published in 1841 as “*Sopra un feto umano tricefalo. Memoria ostetrica ed anatomica*” (Reina, 1841). It concerned a male conjoined triplet with three heads, three arms, and two legs (Figure 4). In his detailed and comprehensive report of the autopsy, Reina mentioned that the two heads on the right side were close together and rested on a single but bifurcated vertebral column, whereas a second vertebral column on the left side carried the third head. In between the third head and the other two, a third arm was present, connected to the trunk with two clavicles and with three bones in the forearm. There were two thoraxes, each with a heart and two lungs, but only one sternum. The heart in the right thorax was twice as large as the one in the left thorax and contained excessive atrial and ventricular tissues. The thoraxes and the abdominal cavity were separated by a single but considerably enlarged diaphragm that almost seemed duplicated. The abdomen was doubled in size contained a markedly enlarged liver and a single spleen, stomach, and pancreas but had a single umbilicus. The intestines were partially duplicated, but there was anal atresia. The shared pelvis was made up of four iliac bones, two sacral bones, two sciatic bones and two pubic bones. The urinary system consisted of a single horseshoe‐shaped kidney, three ureters, and a single bladder. Based on this detailed description and in accordance with the embryonic disk model, it can be concluded that this case concerned a conjoined triplet with the right and middle fetuses forming a *parapagus dicephalus dibrachius* and these two together with the left fetus forming a *parapagus dicephalus tribrachius* (Figure 5a).

Although the two recently described and depicted cases of symmetric conjoined triplets by Rode (Rode et al., 2006) and Athanasiadis (Athanasiadis et al., 2005) are much less detailed than the Reina case (Reina, 1841), it is clear that they represent a different type of conjunction, since both cases featured three heads, four arms, and four legs. Unfortunately, the case from Rode et al. (2006) was only depicted and not described whatsoever. This leaves us with the case described by Athanasiadis et al. (2005) as the sole source of information regarding the internal anatomy in this conjunction type. It concerned a monochorionic female triplet born at 22 weeks of gestation. Prenatal ultrasound and MRI revealed extended intercorporeal connections, with one fetus being ventrally connected at almost right angles to the other two. This peculiar, crossed position was confirmed after termination of pregnancy. A single umbilical cord was found, containing six vessels. Two fetuses had parallel vertebral columns, joined at the sacrum, and shared most of their body, including the internal organs, with exception of the heads. Their shared abdominal cavity and lower sternal ends were ventrally connected with the left side of the abdomen and pleural cavity of the third fetus. This fetus had its own head, extremities, intestinal system, spleen, kidneys, and adrenals but shared a common liver with the other two fetuses. The first two fetuses shared a single heart that must have sustained the circulation in the third fetus as well since the heart of this fetus was severely hypoplastic. Conclusively, this case is to be diagnosed as a conjoined triplet with two fetuses forming a *parapagus dicephalus dibrachius* and a third fetus forming an *omphalopagus* with the other two. The skewed orientation and left‐sided conjunction of the third fetus with the other two, as well as the presence of two hearts, a single liver and single umbilicus, can all be accounted for by the embryonic disk model (Figure 5b). The hypoplasia of the third fetus' heart likely resulted secondarily from circulatory anomalies.

The picture of a conjoined triplet published by Rode et al. (2006) shows resemblance with the case described by Athanasiadis et al. (2005) in the sense that two fetuses, that seem to share a single body with two heads and three arms, are ventrally connected with a third fetus that has its own head and extremities. However, the longitudinal body axes of all three fetuses have the same orientation and the ventral connection between the third fetus and the other two seems to include not only the abdomen but most of the thoracic region as well. This case should therefore be diagnosed as a conjoined triplet with two fetuses forming a *parapagus dicephalus tribrachius* and a third fetus forming a *thoracoileopagus* with the other two. According to the embryonic disk model, this implies that the three fetuses shared a common multichambered heart and two neo‐axially oriented compound livers (Figure 5c).

A possible fourth case of conjoined triplets, that lived for 2 h, was reported to have been born in Samsun, Turkey in the 1950s, with three heads, four arms and four legs (Anonymous., 1954). At autopsy, two hearts, four lungs, and three livers were found. If veritable, this case probably resembled that of Athanasiadis et al., in that it concerned parapagus dicephalus dibrachius conjoined twins and a third fetus forming an omphalopagus with the other two, provided that the reported three livers in fact formed a compound, multilobulated structure.

### 1.3 Historical case descriptions of asymmetric conjoined multiplications: Twins or triplets?

Investigation of the historical literature regarding cases of asymmetric human conjoined multiplications revealed 10 cases, presented here in chronological order and dating back from 1718 till 1983, that all have two particular phenotypic characteristics in common, being the presence of a ventral parasite and (except for one) duplications of facial structures. From an exterior perspective, this pattern of malformations matches not only with asymmetric prosopo‐ and cephalothoracoileopagus conjoined twins but also with asymmetric conjoined triplets. As we will point out, analysis of the internal morphology is crucial to determine which of these diagnoses is correct. Since details on internal morphology were provided in only 7 of these 10 cases, the remaining three cases were left out of further consideration (see Supplementary data S2).

#### 1.3.1 The Trombelli case (1718)

**FIGURE 6:**
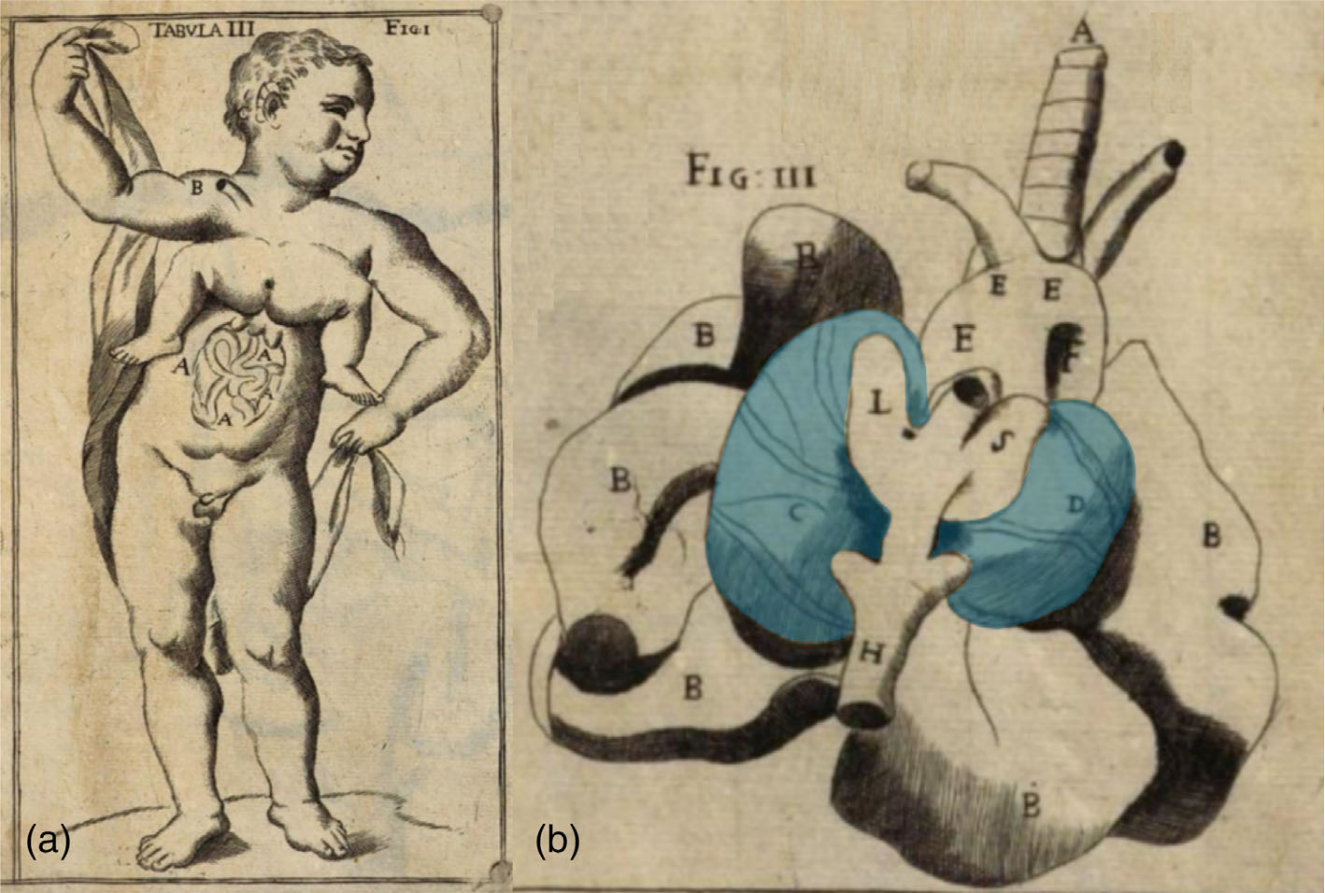
The case described by Trombelli (Vallisneri & Hertz, 1721). (a) Exterior aspect; (b) thoracic organs, including two side‐by‐side located hearts (blue inserts)

In a letter to Giovanni Morgagni, at the time medical professor at the University of Padua, the Bolognese physician Antonio Trombelli described and depicted the autopsy of a male neonate, born in November 1718 at 7 months of gestation, that presented with a ventral parasitic twin and a large abdominal wall defect. Antonio Vallisneri, the predecessor of Morgagni, included this case in his work *Istoria della generazione dell uomo* (Vallisneri & Hertz, 1721). The parasite was attached to the upper part of the autosite's sternum and consisted of a lower body half with an imperforate anus, an empty and divided scrotum, and two normally formed legs, missing only one of the toes (Figure 6a). Examination of the abdomen revealed two livers and a complex of intestinal loops with several duplications, blind ending parts, and connections with the urinary bladders. This included a tortuous, tube‐like structure that protruded from beneath the autosite's right clavicle. The thorax contained both a left‐sided and a right‐sided heart, enclosed by connected pericardial sacs (Figure 6b). Behind the autosite's right ear a rudimentary auricular structure was seen, with two auditory openings. No other facial duplications were reported (Magno et al., 2022).

#### 1.3.2 The Bongiovanni case (1789)

**FIGURE 7:**
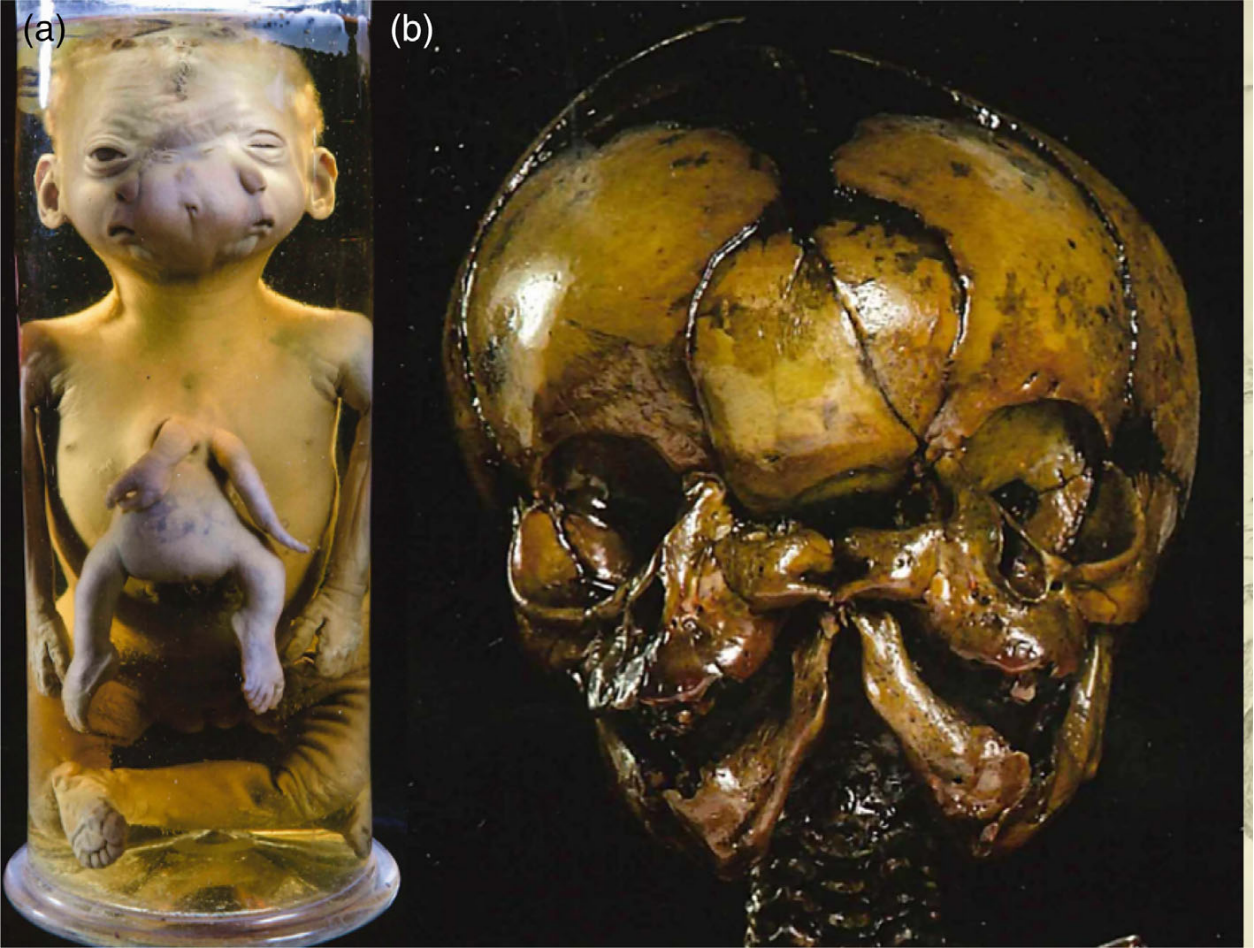
The case described by Bongiovanni (Bongiovanni, 1789). (a). Specimen from the Morgagni Museum of Pathological Anatomy at the University of Padua (Italy) showing the ventral parasite and facial duplications. (b). Dried skull that was extracted and preserved.

This case concerns a wet taxidermic specimen of a female neonate and its dried skeleton (Figure 7a/b). The specimens are part of the collection of the Morgagni Museum of Pathological Anatomy at the University of Padua, Italy (Zanatta, Thiene, Valente, & Zampieri, 2015; Zanatta & Zampieri, 2018) and were described by Zenone Bongiovanni in 1789 (Bongiovanni, 1789). On external inspection the child was well‐formed, except for the presence of an almost completely duplicated face and a ventrally attached parasitic twin. All internal organs of the autosite, including the heart and liver, were of normal and singular morphology, except for the intestines, which were connected to those of the parasite and partially protruding in the autosite's thorax as a result of a diaphragmatic hernia. The parasite consisted of a headless trunk with rudimentary intestines, arms, and legs. Each of autosite's faces consisted of a mouth, a nose with a single nostril, a normally formed outward placed ear and eye, and a small inward placed eye opening. Below the inward placed eyes a protuberance and a slit‐like opening were visible, corresponding with conjunction of zygomatic arches and external auditory meati, as can be concluded from examining the skull (Magno et al., 2022).

#### 1.3.3 The Rosenstiel case (1824)

**FIGURE 8:**
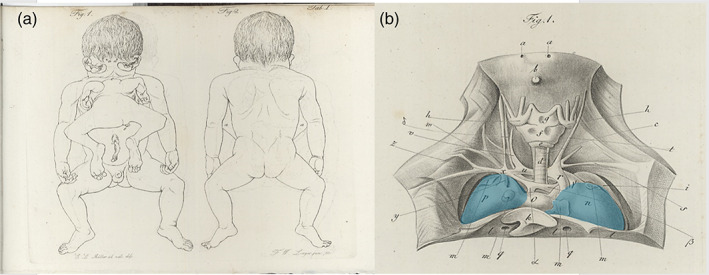
The case described and depicted by Adolf Rosenstiel (Rosenstiel, 1824). (a) Lithograph of the gross specimen. (b) Lithograph of the thoracic anatomy in which two side‐by‐side positioned hearts were found (blue inserts). *Source*: Copies of the original manuscript obtained from the Ghent University Library (Belgium) (BIB.MED.003476/17)

Adolf Rosenstiel described and depicted a male neonate with remarkable facial malformations and a ventrally attached parasitic twin (Rosenstiel, 1824) (Figure 8a). The specimen, which Rosenstiel was granted to dissect, had already been explored previously during which the abdominal organs were removed. He therefore focused his investigations on the cranial and thoracic structures. The facial structures seemed to consist only of two adjacent sets of ears, without any other recognizable rudiments. However, in contrast to the information handed down to Rosenstiel, he did notice all four ears to possess an external auditory meatus and he identified a tiny ocular orifice above both sets of ears. Successive depictions after dissection included the specimens' cardiac morphology. As Rosenstiel described; the left atrium of the right heart was connected to the right atrium of the left heart by means of an additional cavity. The right heart consisted of a single ventricle from which a pulmonary trunk arose that was connected to the brachiocephalic trunk of the left heart (Figure 8b). The parasite lacked a head and thoracic organs but did possess some vertebrae and small but more or less well‐developed extremities.

#### 1.3.4 The Peter case (1844)

**FIGURE 9:**
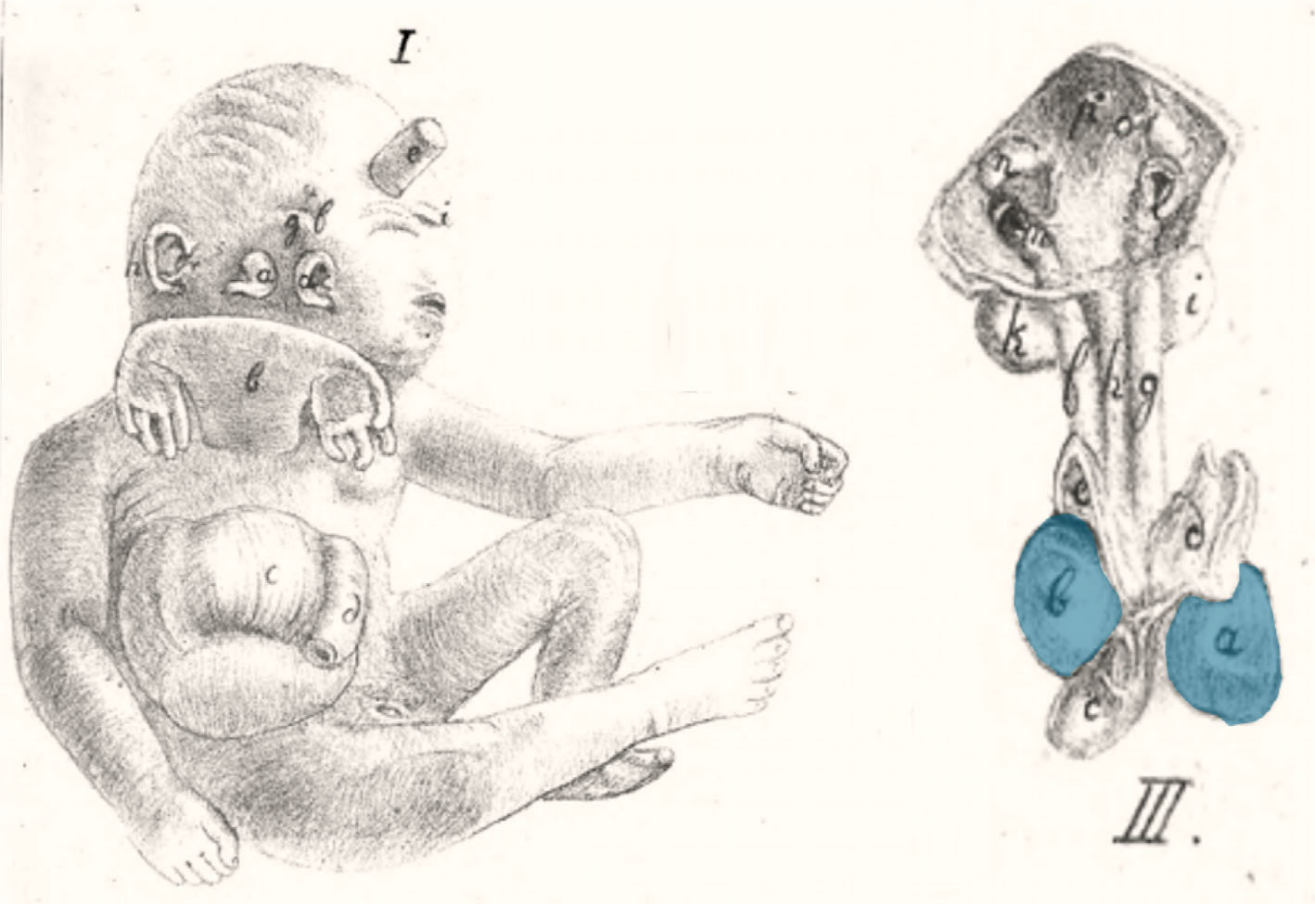
Lithograph of the case described and depicted by Ulrich Peter (Peter, 1844). The accompanying drawing shows the thoracic organs with two side‐by‐side located hearts (blue inserts).

In his theses, Ulrich Peter concerns the case of a female fetus born at 7 months of gestation with a peculiar facial phenotype and an almost cervically inserted ventral parasitic twin (Peter, 1844). Peter's observations mentioned a complete but cyclopic face on the left side of an enlarged head, with two ears and a single palpebral fissure, flanked by four rudimentary eye lids, and a proboscis and a mouth above and below it, respectively. The right side of the head presented with a rudimentary face comprising two ears and an opening that Peter interpreted as an ocular orifice. In the autosite he described the presence of two side‐by‐side located hearts, three lungs, two tracheas, a split liver with two gallbladders and an omphalocele (Figure 9). The ventral parasite consisted of little more than two rudimentary upper extremities.

#### 1.3.5 The Pannullo case (1952)

**FIGURE 10:**
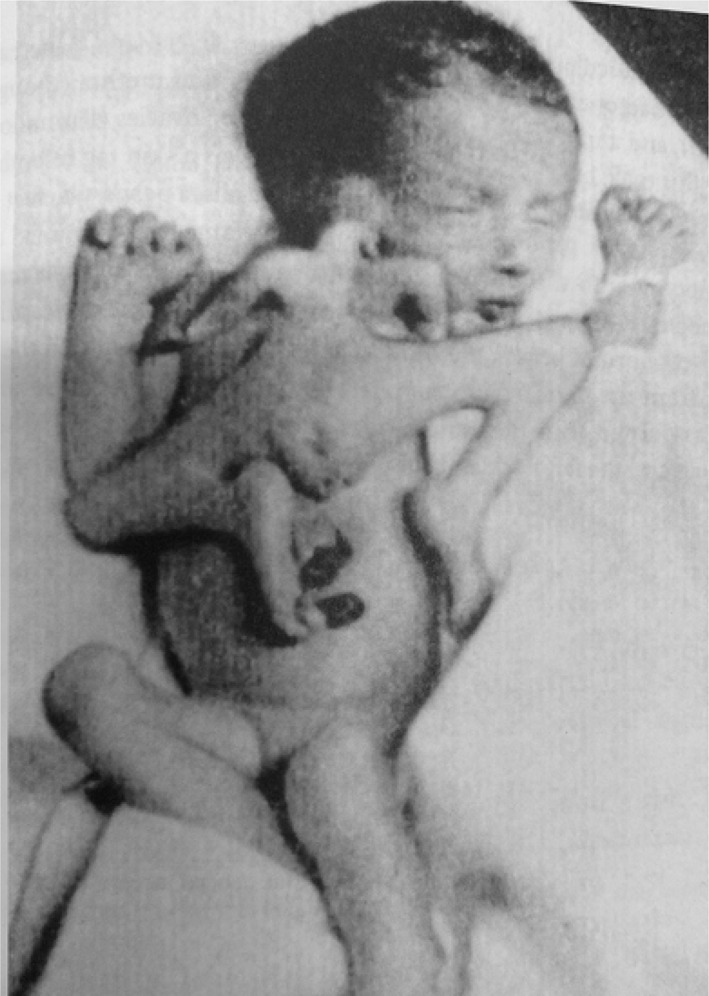
Gross specimen described by Pannulo (Pannullo, 1952). *Source*: Used with permission from *American Journal of Obstetrics and Gynecology*. Publisher: Elsevier. Copyright © 1952 Published by Mosby, Inc.

This case concerns a prematurely born female neonate that was part of a triplet pregnancy and lived for 3.5 days (Pannullo, 1952) (Figure 10). A superiorly inserted ventral parasite was seen that was attached to the right ear and the anterior chest of the autosite through some cervical vertebrae and further consisted of rudimentary arms, fairly well developed legs and normally formed external genitals. The autosite possessed two livers fused at the superior border with two separate gall bladders, two thymuses, two spleens and two pancreases. Additionally, it showed a normal heart on the left and a hypoplastic heart on the right side.

#### 1.3.6 The Stephens case (1982)

**FIGURE 11:**
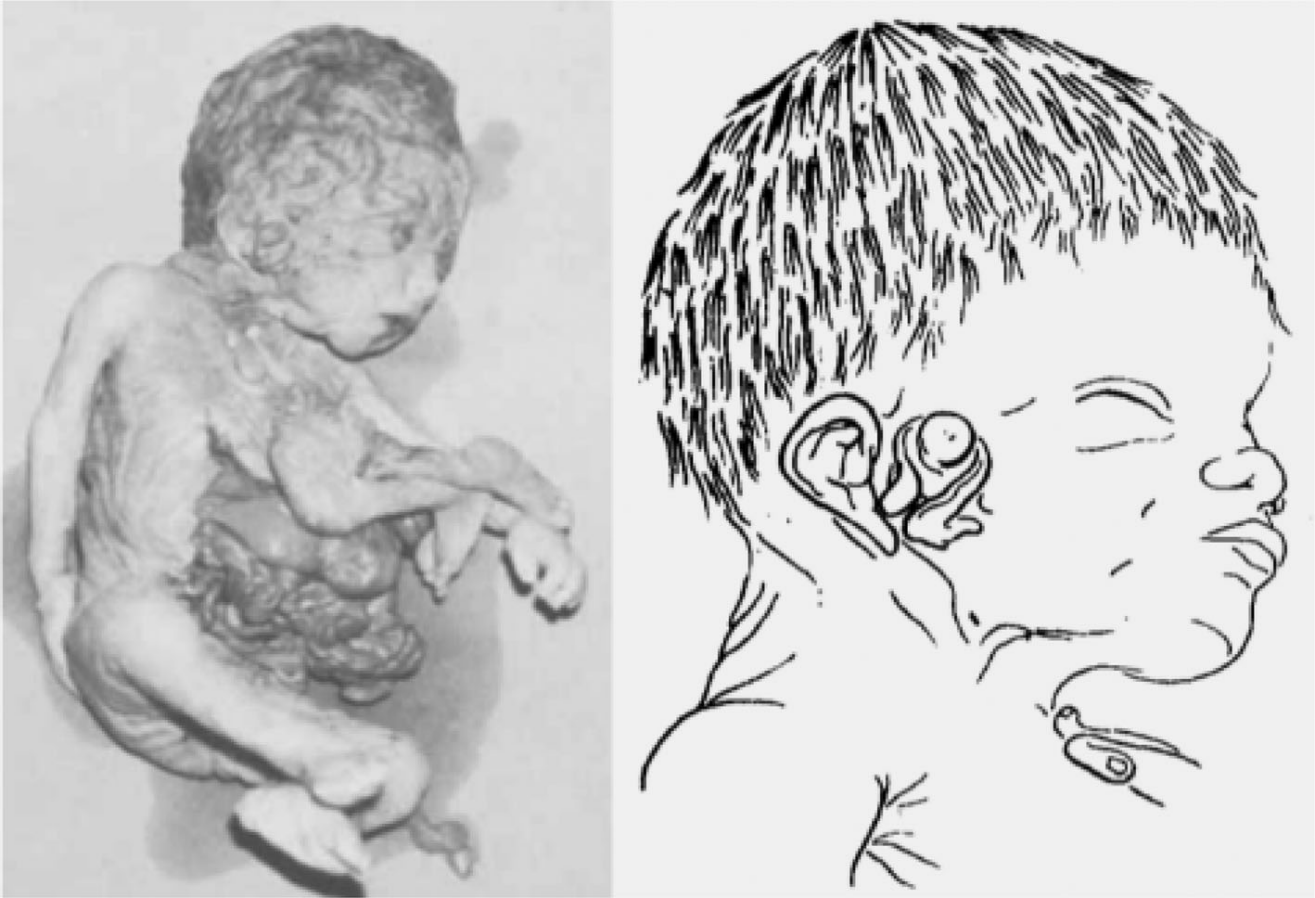
Gross specimen of a male fetus described by Stephens and schematic depiction of its facial morphology (Stephens et al., 1982). *Source*: Used with permission Copyright 1982 Wiley‐Liss, Inc., A Wiley Company

This case concerns a male fetus with a ventrally attached parasite (Stephens, Siebert, Graham, & Beckwith, 1982). On the right side of the autosite's face, a skin tag was present in front of the right ear, which was reminiscent of two fused ectopic and abnormally developed ears (Figure 11). In addition to two fairly well‐formed lower extremities, the parasite had two finger‐like appendages attached to either side of the autosite's upper sternum; there were no vertebrae. The twins shared an omphalocele that mostly contained intestines originating from the autosite but connected with those of the parasite. There was a liver with two large lobes, two stomachs, two spleens associated with the autosite, and one with the parasite. Already showing overlap with the former described cases. A relative normal heart was found on the left side of the autosite's thorax. On the right side, there was a single ventricle with two atria; each heart owned a single outflow tract fusing in the midline and both were located in a single pericardial cavity. A third ventricle located within a separate pericardial sac was associated with the parasitic legs and with the left neck and shoulder of the autosite.

#### 1.3.7 The Chatterjee case (1983)

**FIGURE 12:**
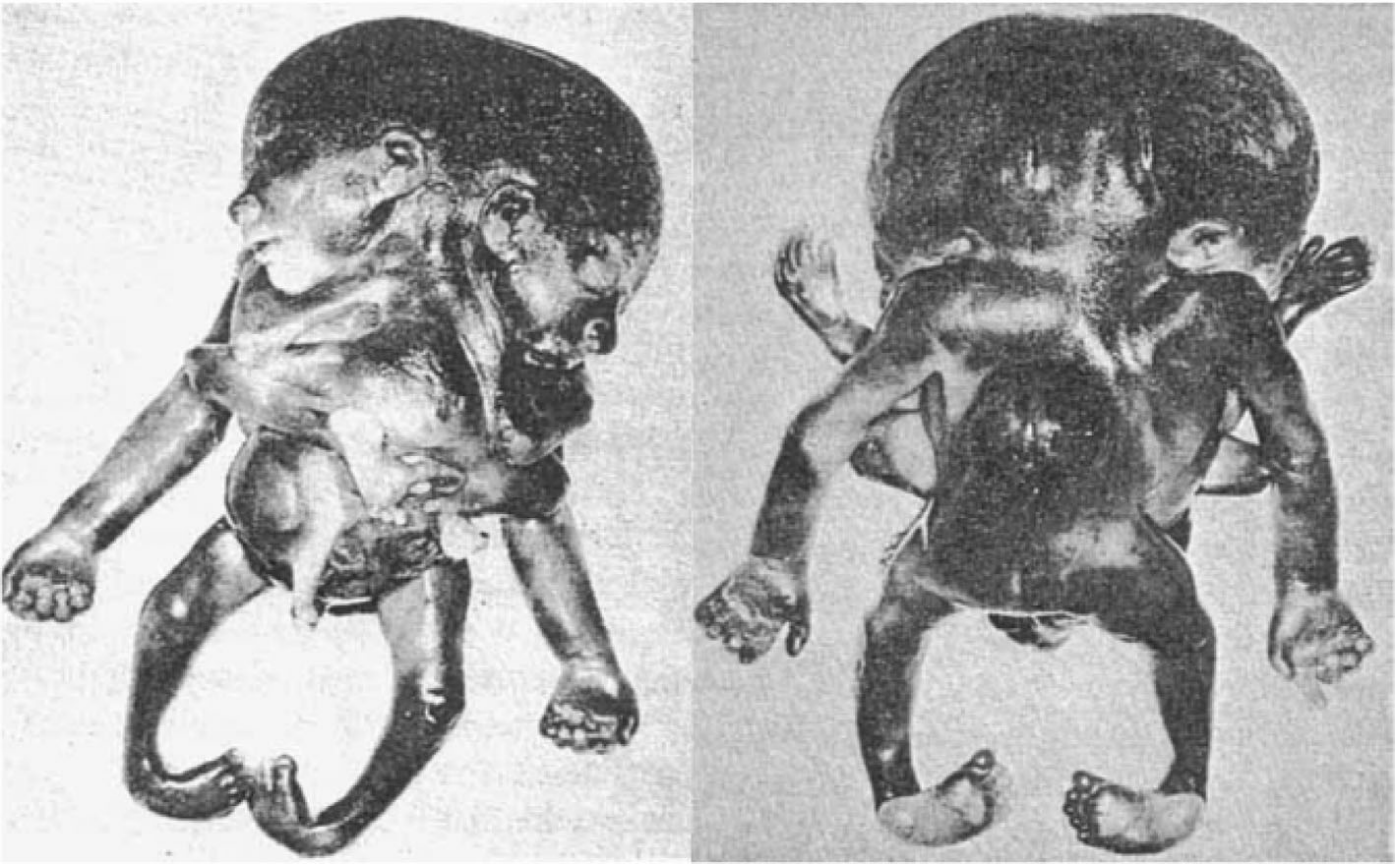
Gross specimen (ventral and dorsal view) described by Chatterjee (Chatterjee et al., 1983). *Source*: Used with permission from John Wiley and Sons, Ltd.

This case was detected by US after elevated serum alpha‐fetoprotein levels were detected at 18 weeks of gestation (Chatterjee, Weiss, Verma, Tejani, & Macri, 1983). US at 23 weeks gestation showed a female fetus with an unusual symmetrical shape of the skull and absent echoes in the midline, together with a thickened fetal body suggestive of a cranial malformation and conjoined twinning; upon which the parents decided for termination of the pregnancy. At autopsy an enlarged head with complete duplication of all facial structures was noticed in addition to a well‐defined ventrally inserted parasite (Figure 12). The autosite had a large thoraco‐lumbar meningomyelocele, and club feet and shared an omphalocele with the parasite. Postmortem angiography showed a single heart with a double aortic arch, each vascularizing one of the upper body halves, and a single descending aorta.

### 1.4 At the heart of the matter

From the embryonic disk models, applied to the cases described by Kaufmann (Kaufmann et al., 2007) and Kastenbaum (Kastenbaum et al., 2009) it can be inferred that asymmetric cephalothoracoileopagus twins have two hearts positioned side‐by‐side, that is, parallel with the plane of conjunction, which concurs with findings of Spencer (2001). On the other hand, as the cases by Athanasiadis (Athanasiadis et al., 2005) and Rode (Rode et al., 2006) demonstrate, conjoined triplets with combined lateral and ventral conjunctions have either two hearts in an antero‐posterior orientation (i.e., perpendicular to the ventral conjunction plane) if the ventral component has an omphalopagus conjunction, or only one (multichambered) heart if the ventral component has a thoracoileopagus conjunction.

**FIGURE 13:**
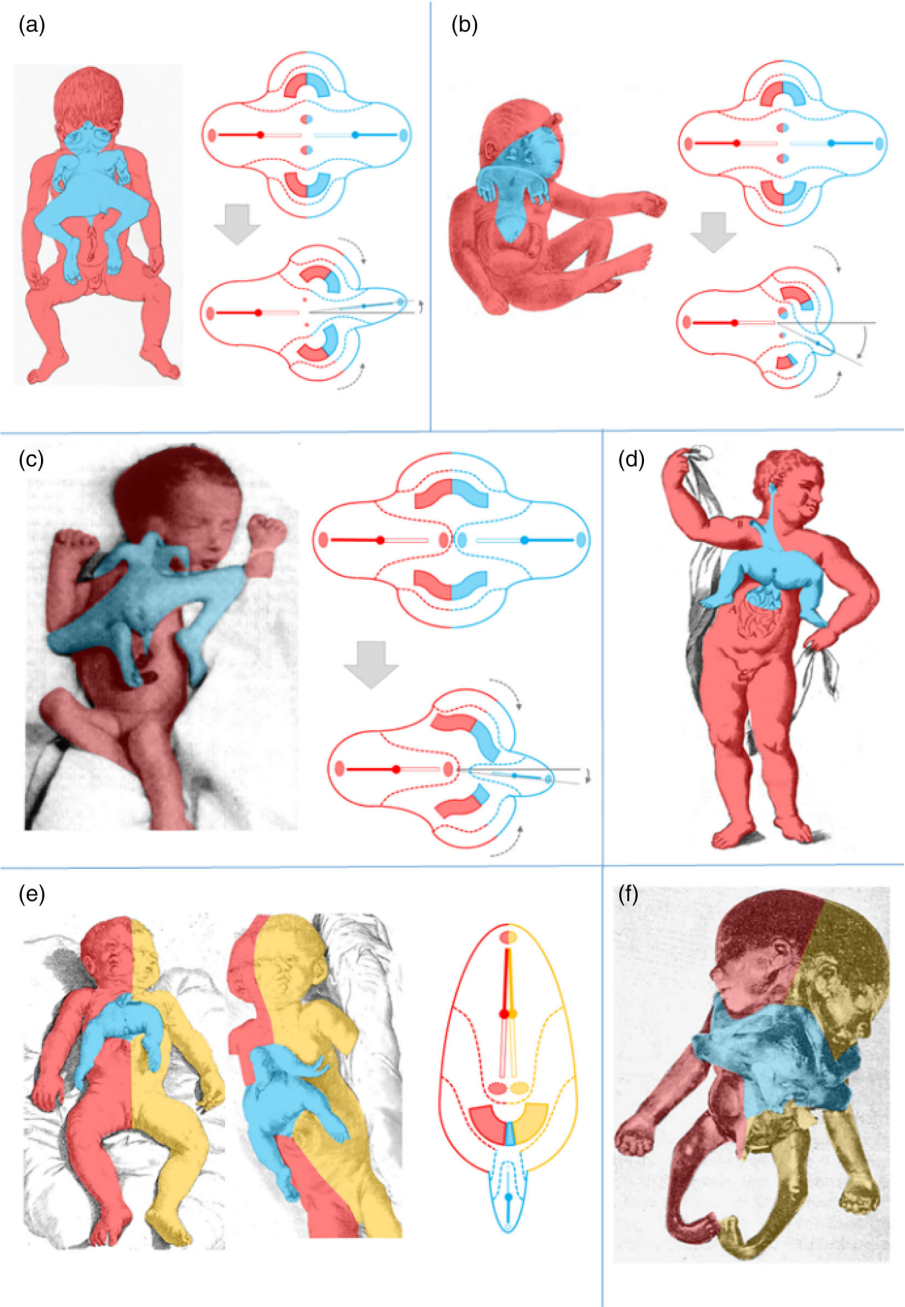
The embryonic disk model applied to 6 of the 10 cases. (a) Rosenstiel case: asymmetric cephalothoracoileopagus conjoined twins. (b) Peter case: asymmetric cephalothoracoileopagus conjoined twins. (c) Pannullo case: asymmetric prosopothoracoileopagus conjoined twins. (d) Trombelli case: asymmetric prosopothoracoileopagus conjoined twins. (e) Bongiovanni case: parapagus diprosopus‐thoracoileopagus conjoined triplet. (f) Chatterjee case: parapagus dicephalus‐thoracoileopagus conjoined triplet (the embryonic disk model of the Pannullo case (c) also applies to the Trombelli case (d), as does the model of the Bongiovanni case (e) to the Chatterjee case (f)).

Returning now to the historical case descriptions it seems reasonable to assume that both the Rosenstiel and Peter cases concern asymmetric cephalothoracoileopagus conjoined twins, since they both featured two side‐by‐side positioned hearts (Figure 13a, b). This is also what is found in the Trombelli case and in the Pannullo case. However, what makes the latter different from the other cases presented here is that there were no true facial duplications but merely a connection between the parasite and the region surrounding the autosite's right ear, leaving the remainder of its face unaffected. We therefore consider this specific morphology to fit best with *asymmetric prosopothoracoileopagus* (Figure 13c). Since the facial duplications in the Trombelli case are confined to a rudimentary auricular structure, we consider this morphology also to represent asymmetric prosopothoracoileopagus (Figure 13d). Moreover, regarding the parasite's fairly well‐developed state, the presence of a connection between the pericardial sacs, as reported by Trombelli, would fit better with prosopothoracoileopagus than with cephalothoracoileopagus, as the embryonic disk models demonstrate.

Conversely, the Bongiovanni case and the Chatterjee case were reported to possess only one heart, which leads to the conclusion that these concern conjoined triplets with two fetuses forming a *parapagus diprosopus* and a third one forming an *asymmetric thoracoileopagus* with the other two. It is not surprising that in both cases this single heart was reported to have a normal appearance, since most diprosopus conjoined twins have a morphologically normal heart and the contribution of the ventral parasite to this heart, if any at all, is probably negligible (Figure 13e,f).

**FIGURE 14:**
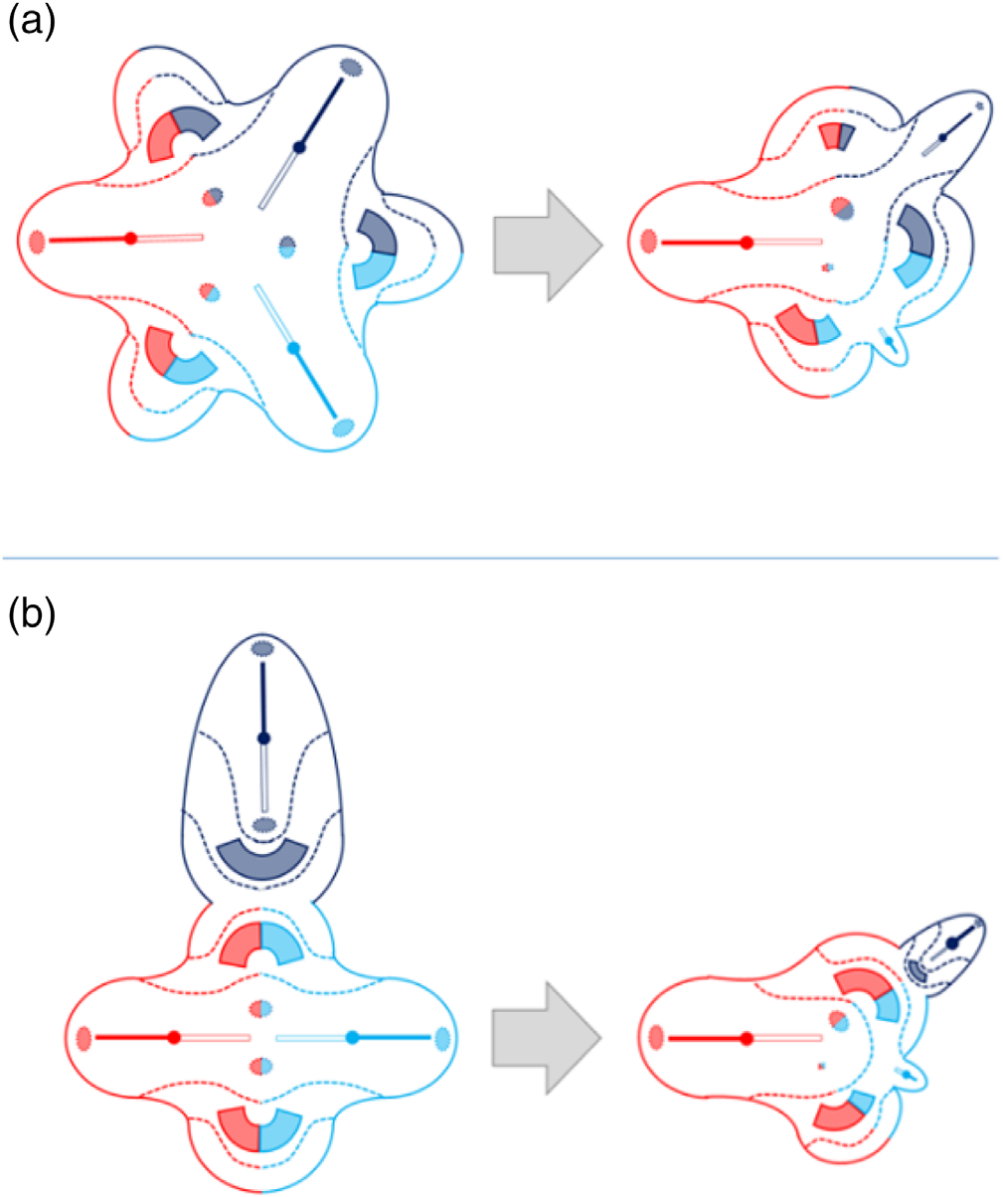
Possible embryonic disk configurations that may explain the Washington case (a) cephalothoracoileopagus‐cephalothoracoileopagus conjoined triplet, (b) cephalothoracoileopagus‐omphalopagus conjoined triplet

Finally, there is the Stephens case, which is unique in that it featured three separate cardiac structures. In our opinion, this is a strong indication of the initial existence of three embryonic primordia, since cardiac duplication in the absence of (conjoined) twinning is an unknown phenomenon in the current scientific literature. Unfortunately, little to no information is presented on the mutual topographic orientation of these hearts and their specific positions in relation to the autosite and the parasite remains speculative. Regarding the type of conjunction, it seems unlikely that it concerns a combination of ventral and lateral components, comparable to the cases of Athanasiadis et al. (2005), Rode et al. (2006), and the Bongiovanni case, as neither of these cases featured more than two hearts. Following the embryonic disk model, the only types of asymmetric conjoined twinning that consistently co‐occur with two separate (compound) hearts are prosopo‐ and cephalothoracoileopagus. Extrapolating from there, this would imply that the Stephens case might be a conjoined triplet with mutual cephalothoracoileopagus conjunctions between each of the three fetuses (Figure 14a). Since the only other type of asymmetric conjoined twinning ever to have been reported with a (rudimentary) parasitic heart was omphalopagus twins (Hesse, 1823; Jain, Borwankar, Parelkar, & Mishra, 2010; Ozcan et al., 2000; Ribeiro et al., 2005; Wirtensohn, 1825) another option would be two fetuses forming a cephalothoracoileopagus and a third one forming an omphalopagus with the other two (Figure 14b).

The most compelling evidence of conjoined duplication or triplication is unequivocal presence of duplicated, respectively, triplicated axial body structures, that is, heads and spinal columns, or other organs, such as the heart, that do not occur multiplied in the absence of twinning. In contrast, redundant spleens, gallbladders, intestines, genitourinary and even facial structures, may also occur in otherwise normal singleton individuals. However, it should be kept in mind that the absence of expected structural triplication does not preclude the diagnosis of conjoined triplets, which can be obscured by the conjunction type and/or parasitic regression. Especially in diprosopus, duplication of any structure other than craniofacial can be indiscernible due to the almost complete overlap of embryonic primordia and the resulting interaction aplasia. This also concerns the vertebral column and the heart and since these structures are usually regressed in parasites it can happen that in a conjoined triplet consisting of a diprosopus with a ventral parasite, such as the Bongiovanni and Chatterjee case, only one vertebral column and one heart are found. Paradoxically―but nonetheless in accordance with the embryonic disk model―, if the autosite in this case would have had two hearts instead of one, conjoined twins (i.e., asymmetric cephalothoracoileopagus) would have been a much more probable diagnosis than conjoined triplets.

## 2 CONCLUSION

Asymmetric conjoined twins and conjoined triplets are exceedingly rare conditions. For the first time, we put these conditions in a developmental perspective by applying the embryonic disk model to seven previously reported case descriptions of asymmetric conjoined multiplications in which triplicated axial structures were lacking, to demonstrate that the number and position of the heart or hearts is of decisive importance in determining the most likely diagnose. In our opinion, the exceptional rarity of conditions like these justifies to obtain as much clinical information as possible, including at least a full body (micro)CT and/or MRI, to further unravel their etiopathogenesis. At the same time, we recommend to avoid any invasive and destructive investigation techniques such as autopsy, and instead, in case of demise, to try and obtain parental permission to store the specimen, either embalmed or frozen, for future diagnostic reference.

## CONFLICT OF INTEREST

No conflict of interest is reported.

## Supporting information


**Appendix 1**: Supporting Information

## Data Availability

Data sharing not applicable to this article as no datasets were generated or analysed during the current study.
