# Supplementary material for: Conjoined twins and conjoined triplets: At the heart of the matter
Source: Birth Defects Res. 2022 Jun 29;114(12):596–610. doi: 10.1002/bdr2.2066 (PMC9546242; doi:10.1002/bdr2.2066)
Supplement: Supplementary file 1 — Appendix 1: Supporting Information [file BDR2-114-596-s001.docx]

Supplementary data

1 Asymmetric cephalothoracoileopagus

To the best of our knowledge, cases convincingly resembling that of Kaufmann (Kaufmann, Comalli Dillon, Cuillier, Lafitte, & Ranjatoelina, 2007) have been reported only twice over the last 250 years. Bordenave (Bordenave, 1776) described and depicted a term female infant, born together with a separate and normally formed twin brother (Fig. S1A). It presented with two opposing faces on a single head, four arms, and two trunks, joint at the chests, one of which was well shaped, the other one vestigial. The latter was described as an irregular shapeless mass that ended in two partially fused legs with normal feet. Bordenave assumed the cause of this malformation to be sought in an accident during conception. Klein (Klein, 1818) described and depicted a prematurely born female that seemed to consist of two infants that were pushed into each other at the level of the heads and chests (Fig. S1B). The head showed two opposing faces, one fairly normal, the other consisting of a central diamond-shaped eye socket, a very small single nostril and two ears joint beneath the chin, without a mouth. One of the trunks was normally formed, the other one had a short, conical shape, without anal or urogenital openings. It ended in a leg-like structure with one nail. Klein realized that these twins were not actually pushed into each other during their development. Rather than that he proposed a mechanism that shows remarkable resemblance with the concepts of neo-axial orientation and interaction aplasia, stating that the divided primordia of both faces joined in the midline to form compound faces, one normal, the other hypoplastic as a result of axial angulation. In both cases the parasite presented with a sirenomelia-like malformation, which contrasts with the case described by Kaufmann. Unfortunately, neither Bordenave nor Klein performed an internal examination.


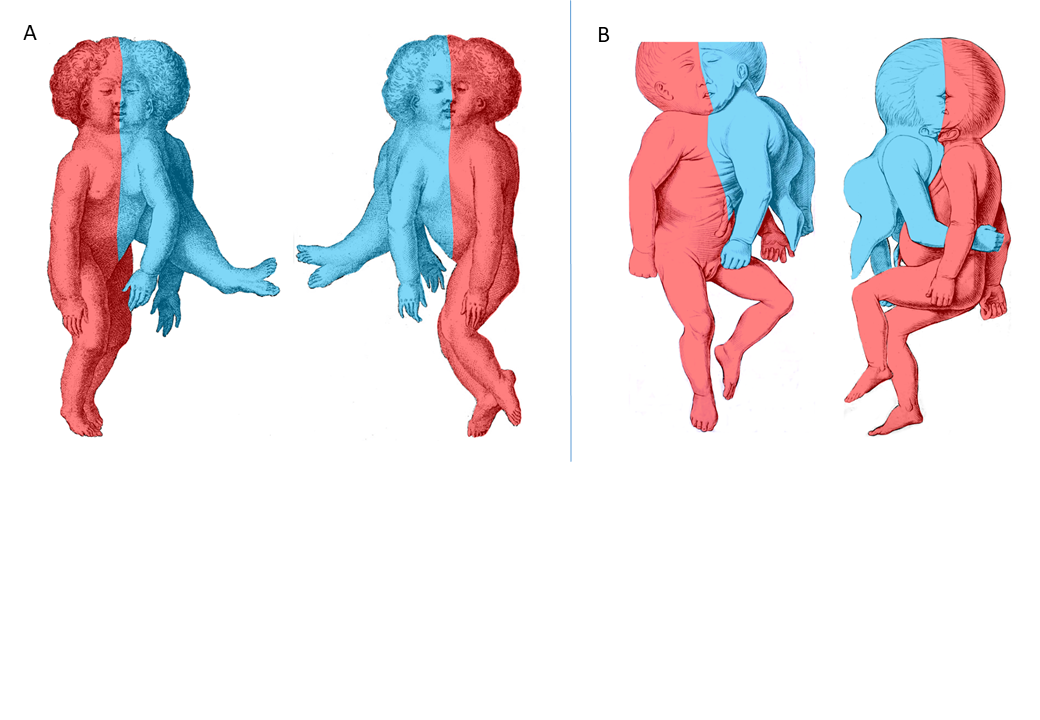


Figure S1. Asymmetric cephalothoracoileopagus. A: case described by Bordenave (1776). B: case described by Klein (Klein, 1818).

2 Additional cases and their analysis

During the process of identifying historical case descriptions of asymmetric anatomical multiplications that could potentially fit the diagnosis of conjoined triplets, we came across three additional cases, the descriptions of which are found below:

*The Biermayer case (1828)*

This case was rediscovered during a recent inventory of the teratological collections of the ‘*Pathologisch-Anatomisches Bundesmuseum*’ (The Federal Pathologic-Anatomical Museum) in Vienna, Austria; also known as the *Narrenturm*. This museum currently comprises more than 50,000 objects and is one of the teratological pinnacles in Europe Patzak and Winter (2013). This wet taxidermic specimen of a new born boy was bought from Joannes Wirtensohn in 1823 and its external characteristics were described and depicted five years later by the museum’s curator Lorenz Biermayer (Biermayer, 1828) (Figs. S2A,B). Apparently, Wirtensohn was interested in these particular cases of asymmetric twinning as he described and depicted two other parasitic twins in his dissertation from 1825 (Wirtensohn, 1825). However, his dissertation lacked any information about this particular specimen that he had sold two years earlier. In addition to a ventrally attached parasitic twin, Biermayer mentioned the autosite to have a broadened head with complete facial duplication, although both faces showed profound and symmetric malformations, including a single but large central palpebral fissure, a small mouth and absence of nasal structures. Each face was flanked by two ears. The autosite’s trunk, as well as its neck, was broad and stubby and showed a lumbar myelomeningocele, hypoplastic male genitalia, anal atresia and club feet. The headless parasite consisted of a trunk with small but well-formed extremities. Both twins shared an omphalocele. Although Biermayer abstained from internal inspection, the skeleton and internal organs were taken out either before or after he acquired the specimen from Wirtensohn; the present whereabouts of this extraction remains unknown. In the last paragraph of his treatise Biermayer reflects on the similarities between this specimen and that of a girl born with a ventrally attached parasitic twin, one head, two ears and three faces. The middle one of these faces consisted of two minute palpebral fissures and a poorly formed nose, mouth and chin. Although no reference is stated, the details of Biermayer’s description leave no doubt that this concerns the Bongiovanni case.


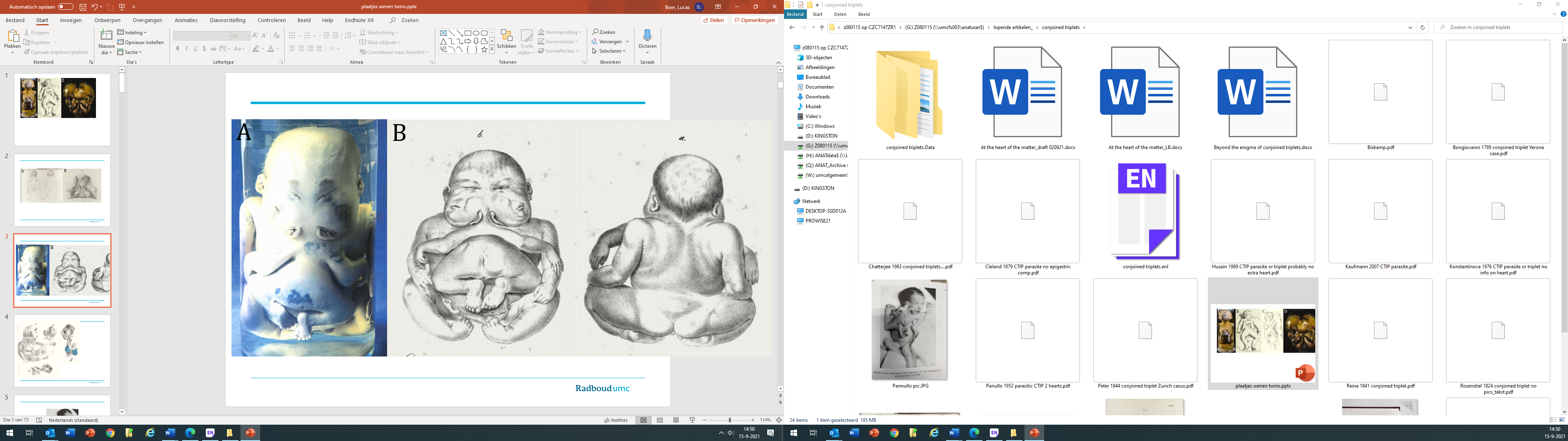


Figure S2. The case described and depicted by Lorenz Biermayer (Biermayer, 1828). A. Photograph of the wet taxidermy specimen from the *Narrenturm* collections in Vienna (Austria). B. Lithographs of the same specimen.

*The Ahlfeld case (1880)*

This case concerns a specimen described and depicted by Friedrich Ahlfeld (Ahlfeld, 1880) presenting with facial duplications and malformations and a superiorly inserted well-developed ventral parasite (Fig. S3). This case was part of the anatomical collection originated from the “*Entbindung Schule*” (Childbirth school) currently maintained by the *Universitätsklinikum* in Leipzig (Germany). Unfortunately, the specimen is no longer part of the collection and the only information available is extracted from Ahlfeld’s brief description in which nothing is mentioned about its internal organs. He described the better developed left face as affected by cyclopia and considered the position of the four ears to be comparable with the Rosenstiel case, in that the two inward placed ears belong to the parasite and the outer ones to the autosite. The parasite showed four well-developed extremities and genital organs.


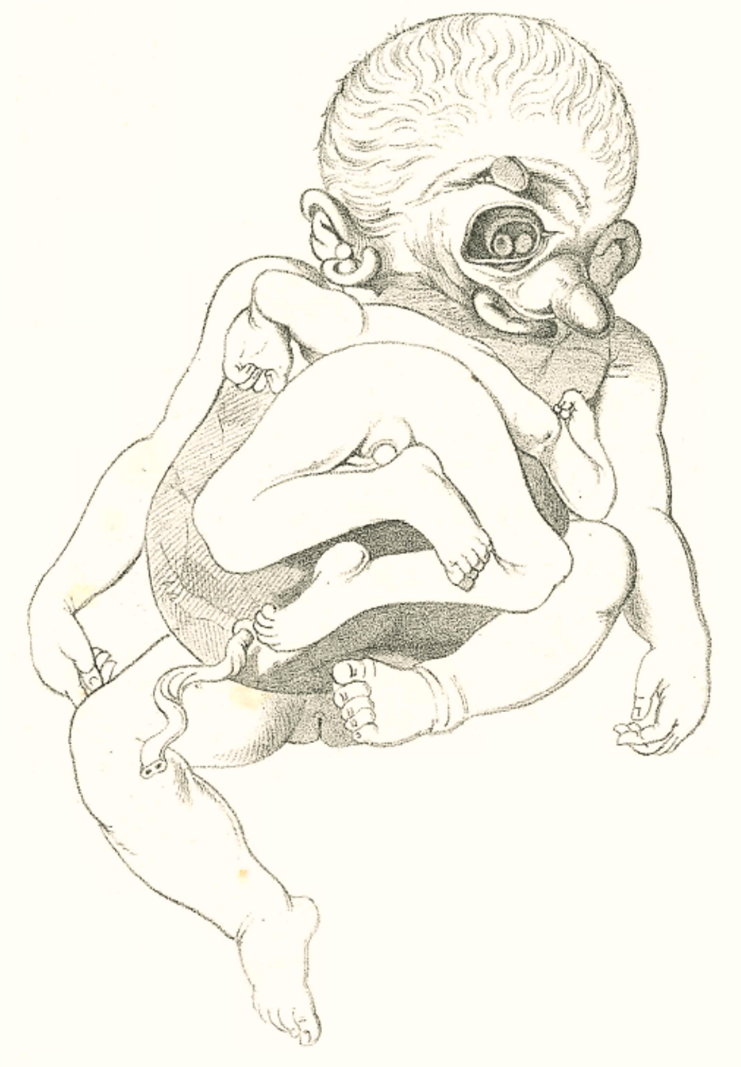


Figure S3. Lithograph of the case described and depicted by Friedrich Ahlfeld (Ahlfeld, 1880) showing asymmetric facial morphology, a well-defined epigastric parasite and an omphalocele.

*The Konstantinova case (1976)*

This case concerns a male neonate with facial duplications and a superiorly inserted ventral parasite, which lived for four days (Konstantinova, 1976) (Fig. S4). In addition to a normal right sided mouth, nose, ear and eye on the right side of the face, the left side showed a partially duplicated eye, a rudimentary mouth and a normally formed ear. The parasite had four extremities and its abdominal cavity appeared to communicate with that of the autosite and contained a horseshoe kidney, urogenital organs and a rudimentary colon. No information concerning its thoracic organs was reported.


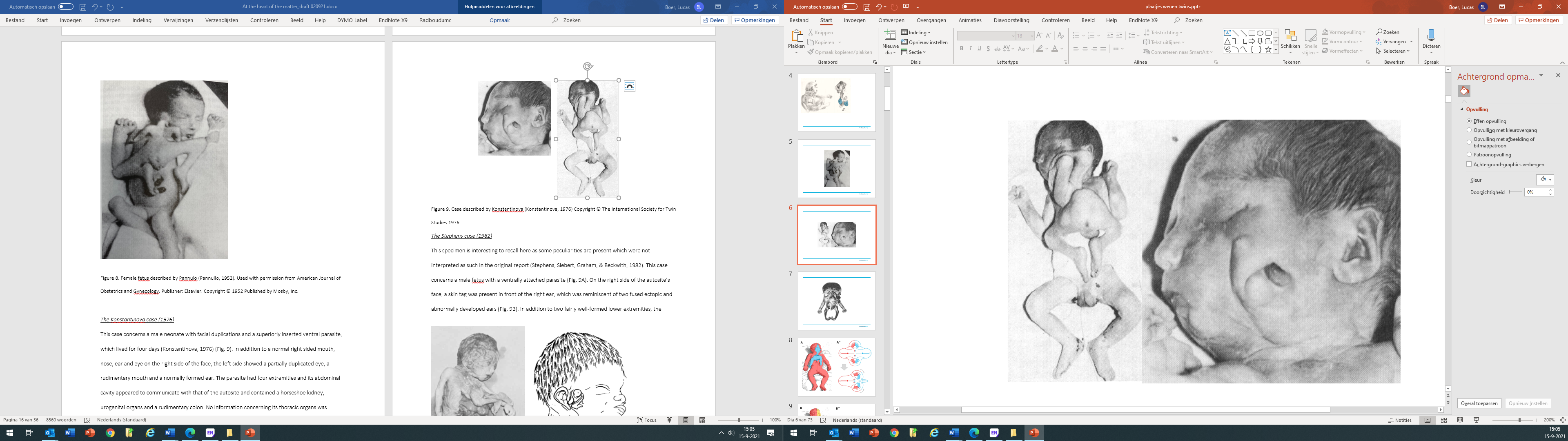


Figure S4. The case described by Konstantinova (Konstantinova, 1976) Copyright © The International Society for Twin Studies 1976.

*Analysis*

Regarding the above cases, the final diagnosis presently remains elusive, since no information concerning the internal organs, i.p. the heart(s), was recorded. Moreover, the presence of (bilateral) midfacial hypoplasia does not make one diagnosis more likely than the other, since this aspect is seen in both asymmetric conjoined twins as well as in asymmetric conjoined triplets. As for Biermayer’s report, it mentions the presence of a lumbar meningocele, anal atresia and malformed external genitals in the autosite, and a shared omphalocele, possibly containing internal organs of both autosite and parasite. This combination is quite suggestive of OEIS complex, a condition known to occur frequently in ventrally conjoined twins, in particular in cephalothoracoileopagus (Boer, Schepens-Franke, Winter, & Oostra, 2021). On the other hand, the parallels between the Biermayer case and the triplet described by Chatterjee, including a shared omphalocele and an autosite with a broadened thorax, a lumbar meningo(myelo)cele, and clubfeet, are intriguing. Regarding the Ahlfeld and Konstantinova case, the available information is inconclusive for either of the diagnoses.

References

Ahlfeld, F. (1880). *Die Missbildungen des Menschen.*: F.W. Grunow.

Biermayer, L. (1828). *Das kais. kön. pathologische Musaeum im allgemeinen Krankenhause zu Wien*: Strauß.

Boer, L. L., Schepens-Franke, A. N., Winter, E., & Oostra, R. J. (2021). Characterizing the coalescence area of conjoined twins to elucidate congenital disorders in singletons. *Clin Anat*. doi:10.1002/ca.23725

Bordenave, T. (1776). Description d'un enfant monstrueux né a terme, ayant deux visages sur une seule tete, et deux corps reunis superieurement, l'un bien et l'autre mal conformés. *Mem. de l'Acad. des sc. de Paris*, 697-699. .

Kaufmann, E., Comalli Dillon, K., Cuillier, F., Lafitte, A., & Ranjatoelina, A. (2007). Abnormal triplet pregnancy. from The fetus net

Klein. (1818). Beschreibung einer Missgeburt. In: Deut Arch für Phys 4: 551-559.

Konstantinova, B. (1976). Morphological and cytogenetic studies on conjoined twins. *Acta Genet Med Gemellol (Roma), 25*, 55-58. doi:10.1017/s0001566000013830

Patzak, B., & Winter, E. (2013). [Karl Alfons Portele, Pathologist and first director of the Federal Pathologic-anatomical Museum Vienna]. *Wien Med Wochenschr, 163*(13-14), 322-326. doi:10.1007/s10354-013-0210-8

Wirtensohn, J. (1825). *Duorum monstrorum duplicium humanorum descriptio anatomica*: Maurer.
